# BCOR::CREBBP fusion in malignant neuroepithelial tumor of CNS expands the spectrum of methylation class CNS tumor with BCOR/BCOR(L1)-fusion

**DOI:** 10.1186/s40478-024-01780-5

**Published:** 2024-04-18

**Authors:** Azadeh Ebrahimi, Andreas Waha, Jens Schittenhelm, Georg Gohla, Martin U Schuhmann, Torsten Pietsch

**Affiliations:** 1 Institute of Neuropathology, DGNN Brain Tumor Reference Center, University of Bonn, Venusberg-Campus 1, D-53127 Bonn, Germany; 2 Institute of Neuropathology, University Hospital of Tübingen, Tübingen, Germany; 3 Department of Diagnostic and Interventional Radiology, University Hospital of Tübingen, Tübingen, Germany; 4 Department of Neurosurgery, University Hospital of Tübingen, Tübingen, Germany

**Keywords:** Methylation class CNS tumor with BCOR/BCOR(L1)-fusion, BCOR::CREBBP fusion, Neuroepithelial tumor of CNS

## Abstract

Methylation class “CNS tumor with BCOR/BCOR(L1)-fusion” was recently defined based on methylation profiling and tSNE analysis of a series of 21 neuroepithelial tumors with predominant presence of a BCOR fusion and/or characteristic CNV breakpoints at chromosome 22q12.31 and chromosome Xp11.4. Clear diagnostic criteria are still missing for this tumor type, specially that BCOR/BCOR(L1)-fusion is not a consistent finding in these tumors despite being frequent and that none of the Heidelberger classifier versions is able to clearly identify these cases, in particular tumors with alternative fusions other than those involving BCOR, BCORL1, EP300 and CREBBP. In this study, we introduce a BCOR::CREBBP fusion in an adult patient with a right temporomediobasal tumor, for the first time in association with methylation class “CNS tumor with BCOR/BCOR(L1)-fusion” in addition to 35 cases of CNS neuroepithelial tumors with molecular and histopathological characteristics compatible with “CNS tumor with BCOR/BCOR(L1)-fusion” based on a comprehensive literature review and data mining in the repository of 23 published studies on neuroepithelial brain Tumors including 7207 samples of 6761 patients. Based on our index case and the 35 cases found in the literature, we suggest the archetypical histological and molecular features of “CNS tumor with BCOR/BCOR(L1)-fusion”. We also present four adult diffuse glioma cases including GBM, IDH-Wildtype and Astrocytoma, IDH-Mutant with CREBBP fusions and describe the necessity of complementary molecular analysis in “CNS tumor with BCOR/BCOR(L1)-alterations for securing a final diagnosis.

**Supplementary Information:**

The online version contains supplementary material available at 10.1186/s40478-024-01780-5.

## Introduction

Methylation class “CNS tumor with BCOR/BCOR(L1)-fusion” was recently defined based on methylation profiling of a series of 21 neuroepithelial tumors with predominant presence of a BCOR fusion and/or characteristic CNV breakpoints at chromosome 22q12.31 and chromosome Xp11.4 [32]. Clear diagnostic criteria are still missing for this tumor type, specially that BCOR/BCOR(L1)-fusion is not a consistent finding in these tumors despite being frequent and that none of the Heidelberger classifier versions is able to clearly identify these cases, in particular tumors with alternative fusions other than those involving BCOR, BCORL1, EP300 and CREBBP. In this study, we introduce a BCOR::CREBBP fusion in an adult patient with a right temporomediobasal tumor and methylation class “CNS tumor with BCOR/BCOR(L1)-fusion”. This study presents a comprehensive literature review for additional inputs to this methylation class and explores the genetic alterations of BCOR, BCORL1 and their typical fusion partners EP300, TRAP1, CREBBP, and L3MBTL2 in the repository of 23 published studies on neuroepithelial brain Tumors.

## Case presentation

**Fig. 1:**
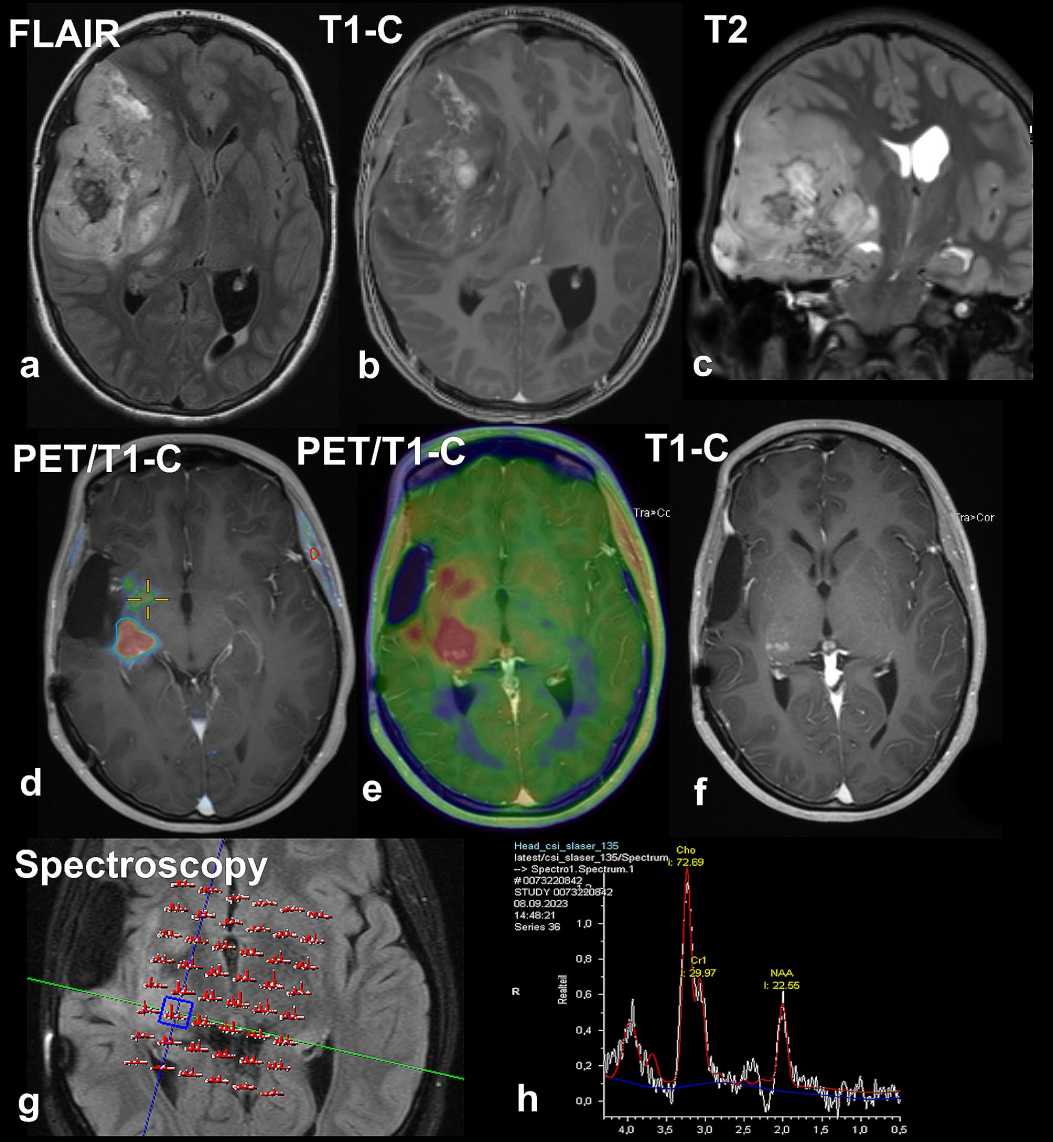
Radiological presentation of primary and recurrent tumor. The primary tumor was an inhomogeneous right temporomediobasal mass with slight contrast enhancement (T1-C) and consecutive shift of midline structure (**a-c**). Thirty months later, the PET/MRI and MR spectroscopy findings were clearly indicative of tumor recurrence. 18 F-fluoroethyl-tyrosine PET showed increased tracer uptake in the contrast-enhanced areas of the right basal ganglia and thalamus (**d-f**), and MR spectroscopy showed a corresponding tumor profile with increased choline and decreased N-acetylaspartate peaks (**g-h**)

**Fig. 2:**
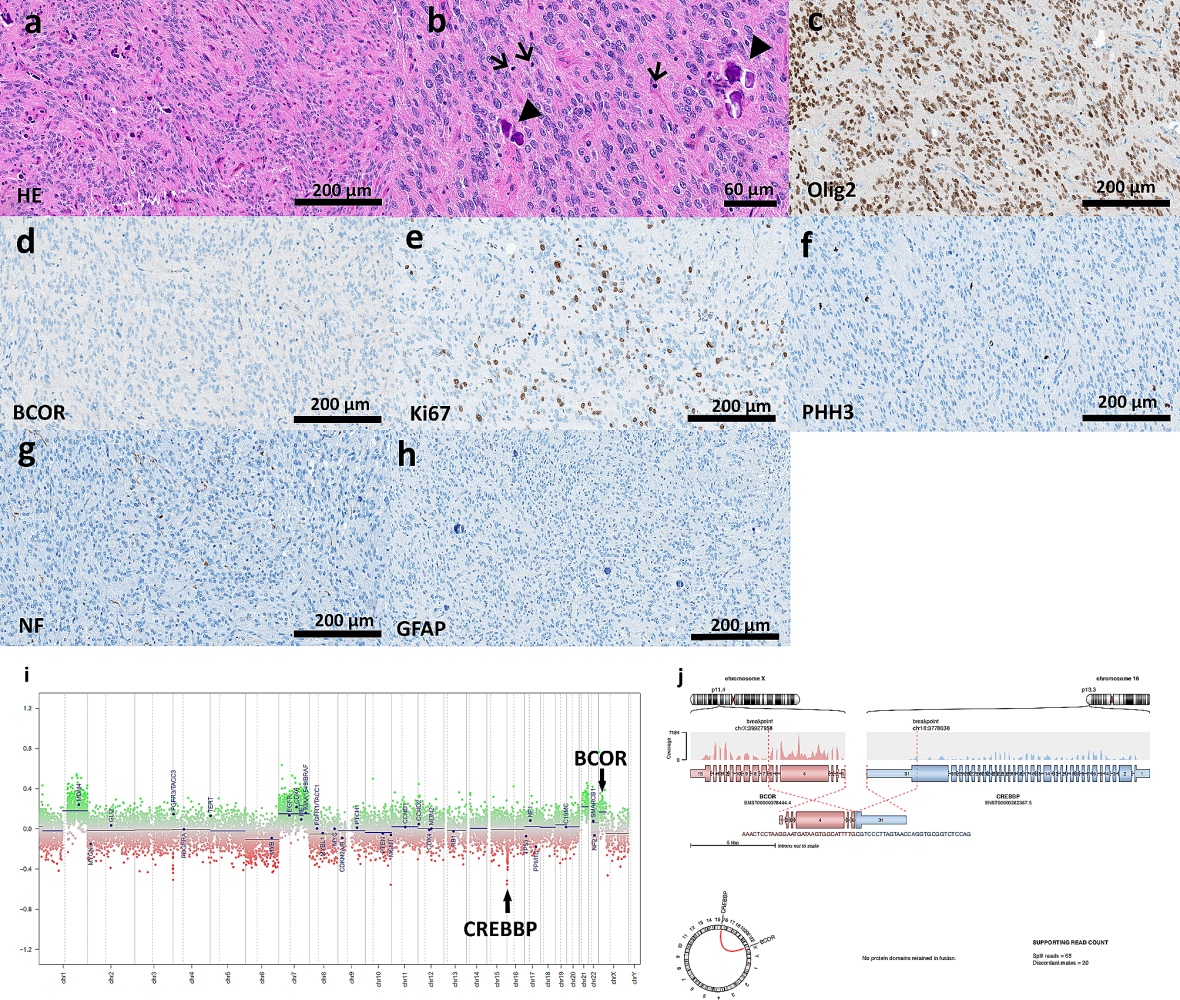
Histological, immunohistochemical and genetic/epigenetic features of the tumor. **(a)** Histological exam revealed a hypercellular neuroepithelial tumor with ependymoma-like histological pattern including perivascular pseudorosettes, focal calcifications **(a, b)**, and a high proliferation activity **(e)** with frequent mitotic figures **(b, f)**. Olig2 was expressed in tumor cells and BCOR was negative **(c, d)**. NF staining confirmed a diffuse growth pattern **(g)**. GFAP was negative **(h)**. **(j)** RNA-based next generation sequencing revealed a BCOR::CREBBP fusion with breakpoints in intron 6 of BCOR and exon 31 of CREBBP. **(i)** The copy number profile showed focal alterations on chromosomal regions 16p13.3 and Xp11.4 representing the CREBBP and BCOR gene locations

Our case was a 14 years old girl with a large inhomogeneous right temporomediobasal mass with slight contrast enhancement and consecutive shift of midline structures in MRI (Fig. 1). After gross total resection, histological exam showed a hypercellular neuroepithelial tumor with ependymoma-like histological pattern including perivascular pseudorosettes, focal calcifications, and a high proliferation activity (Fig. 2). IDH1/2 pyrosequencing and pTERT Sanger sequencing showed wildtype sequences. Methylation profiling showed a significant calibrated score of 0.99 in v12.5 and v12.8 versions of Heidelberger brain tumor classifier and lower calibrated score of 0.79 in v11.b4 for methylation family class “CNS tumor with BCOR alteration”. While the versions 12.5 showed a calibrated score of 0.65 for the subtype “CNS tumor with BCOR/BCOR(L1)-fusion”, the versions 12.8 showed a discrepant result with calibrated score of 0.91 for the subtype HGNET-BCOR-ITD; however, PCR analysis showed no BCOR-ITD. RNA-based NGS analyses revealed a BCOR::CREBBP fusion (Fig. 2). The BCOR::CREBBP fusion was predicted to be out-of-frame, creating a premature stop codon at codon 2337 within the CREBBP segment. It was a persistent finding in 850 K-methylation analysis with corresponding copy number alterations at chromosomal regions 16p13.3 and Xp11.4 representing the CREBBP and BCOR gene locations (Fig. 2). The patient underwent proton radiation and adjuvant temozolomide therapy. Thirty months later the patient had a tumor recurrence and underwent a partial resection (Fig. 1). At the last follow up 32 months after the first diagnosis, the patient was alive with no evidence of recurrence or progress.

## Discussion

Pathogenic alterations of BCOR have been reported in various tumor types including HGNET-BCOR-ITD, CNS tumor with BCOR/BCOR(L1)-fusion, endometrial stromal and soft tissue sarcomas, primitive myxoid mesenchymal tumors of infancy and undifferentiated round cell sarcoma. Regarding CNS tumor with BCOR/BCOR(L1)-fusion, beside the original publication very few cases have been reported in the literature [12, 25, 26, 29, 33, 34]. The core characteristic of “CNS tumor with BCOR/BCOR(L1)-fusion” is defined as predominant presence of a BCOR fusion with often EP300 as the fusion partner and/or characteristic CNV as mentioned before. In the primary study, occasional cases presented a BCOR stop mutation or MEAF6::CXXC5 fusion instead of the typical BCOR fusions [32].

In a comparative systematic review of the literature, we found 35 published cases of neuroepithelial CNS tumors that either carried a BCOR/BCOR(L1)- or other related fusions, or had a methylation profile compatible with CNS tumors with BCOR/BCOR(L1)-fusion (Supplementary Table 1). The majority of reported cases are supratentorial, but cerebellar localization occurs occasionally. Most of the patients are of pediatric or young adult age. However, this tumor occurs in a wide age range (5–72) with 55% of the cases under 40 and 85% under 50 years of age. The tumor is typically T1-hypointense, T2-hyperintense and can be well circumscribed or diffusely infiltrating both in radiological and in histological exam. Contrast enhancement might be present at the first manifestation. The archetypical histomorphology includes an oligodendroglial or ependymoma-like histological pattern with a delicate ‘chicken-wire’ vasculature and perivascular pseudorosettes. Focal calcifications are frequent and occasionally microcystic/ myxoid changes may be present. The tumor cells show round to oval nuclei with finely speckled chromatin and mild to moderate atypia as well as prominent glial processes. Malignancy features such as necrosis, frequent mitoses and microvascular proliferations might be present at the first manifestation. With the exception of Olig2, which is frequently positive in these tumors, other glial markers are variably expressed. Nuclear expression of BCOR is also variable and cannot be used as a surrogate marker. Although, within the reported cases, the majority did not show a positive nuclear expression of BCOR (15 of 25), 10 cases still showed a nuclear expression of BCOR (Supplementary Table 1). Our case was negative for BCOR. Similarly, the BCOR staining cannot differentiate between HGNET-BCOR-ITD and CNS tumor with BCOR/BCOR(L1)-fusion, since a majority of HGNET-BCOR-ITD also show a nuclear BCOR expression [11]. BCOR-ITD is not present in any of the CNS tumors with BCOR/BCOR(L1)-fusion and this helps differentiating from HGNET-BCOR-ITD. EP300::BCOR is the typical fusion found in these tumors with the common breakpoints exon 31 for EP300 and exon 4–7 for BCOR. Alternative fusions have been reported such as MEAF6::CXXC5, CREBBP::BCORL1, EP300::BCORL1, and BCOR::L3MBTL2 among tSNE-defined cases [2, 32].

We found a reported BCOR::CREBBP fusion in a neuroepithelial tumor with breakpoints in exon 4 of BCOR and exon 31 of CREBBP in the literature [22]. However, this study did not perform a methylation profiling at the time and the tumor did not show archetypical histopathological features of CNS tumors with BCOR/BCOR(L1)-fusion.

Besides, five additional inputs were found after searching for alterations in BCOR, BCORL1, EP300, TRAP1, CREBBP, and L3MBTL2 in the repository of 23 published studies on neuroepithelial brain Tumors including 7207 samples of 6761 patients (Supplementary Fig. 1) [3–10, 13–17, 19–21, 23, 24, 27, 28, 30, 31, 35]. These cases showed further related fusions including EP300::BCOR; BCOR::L3MBTL2; BCOR::EP300; BCOR::TRAP1; CREBBP::GOLGA6L2; CREBBP::ENPP7P14; CREBBP::LCMT1 (Supplementary Tables 1 and 2). Two of the cases with CREBBP::GOLGA6L2 and CREBBP::LCMT1 fusions fulfilled the molecular criteria for GBM, *IDH* wildtype including + 7/-10 signature and homozygous deletion of *CDKN2A/B* as well as *IDH* wildtype status. One case with CREBBP::ENPP7P14 fusion fulfilled the molecular criteria for astrocytoma, IDH-mutant including *IDHR132H*-mut, *TP53*-mut, *ATRX*-mut, no 1p/19q. One case with three fusions (EP300::BCOR; BCOR::L3MBTL2; BCOR::EP300) was described as a neuroepithelial tumor with small spindle cell pattern and spongioblastoma polar-like or ependymoma-like areas, calcifications, chicken-wire vasculature, necrosis, high mitotic activity and seemed to fit the archetypical histological pattern of CNS tumors with BCOR/BCOR(L1)-fusion. However, no methylation-based classification was available for this tumor. One case with BCOR::TRAP1 fusion was diagnosed as diffuse glioma grade 4 and showed mutations on SETD2 C495Lfs*6, PTPN11 A72V, PTPN11 E76G, TP53 E258K and a CDKN2A/B homozygous deletion but could not be further classified (Supplementary Table 1).

The BCOR::CREBBP fusion in our case was predicted to be out-of-frame, resulting in no chimeric transcript and potentially leading to a loss of function of BCOR. BCOR plays a role in pluripotency maintenance, differentiation induction and cell fate determination via regulation of histone methylation through its PCGF Ub-like fold discriminator (PUFD) domain. PUFD is an essential domain for tumor suppressor function of BCOR [18]. Loss of BCOR function is associated with tumorigenesis [1]. On the other hand, the BCOR gene is located on the X-chromosome, available as only one allele, so that the fusion in the index case would potentially lead to a complete loss of putative tumor-suppressor activity mediated by the PUFD. Out-of-frame BCOR::CREBBP fusion has already been reported in malignant diffuse glioma of pediatric age, potentially a supporting evidence against a stochastic event [22].

## Conclusion

CNS tumor with BCOR/BCOR(L1)-fusion seems to be an independently existing tumor type with archetypical histological and molecular features, however, consistent diagnostic criteria are still missing. These tumors frequently show oligodendroglial or ependymoma-like morphology with a delicate ‘chicken-wire’ vasculature and perivascular pseudorosettes, focal calcifications, occasionally microcystic/myxoid changes and malignancy features such as necrosis, frequent mitoses and microvascular proliferations, as well as frequent Olig2 expression. None of the Heidelberger classifier versions is able to clearly identify the cases, especially tumors with alternative fusions other than those involving BCOR, BCORL1, EP300 and CREBBP. Nuclear expression of BCOR is variable and cannot be used as a surrogate marker. Nevertheless, lack of BCOR-ITD helps differentiating them from HGNET-BCOR-ITD. Knowing that adult diffuse gliomas such as GBM, *IDH*-Wildtype and Astrocytoma, *IDH*-Mutant may present CREBBP fusions, it is of utmost importance to take additional molecular findings and the histomorphology into account while interpreting the results. Interpretation of survival data is recommended to be postponed to larger cohorts. Published cases in the literature can be used as a guide for individual decision making for patients.

### Electronic supplementary material

Below is the link to the electronic supplementary material.

Supplementary Material 1

Supplementary Material 2

Supplementary Material 3

## Data Availability

Data are available from the corresponding author on reasonable request.
