# Supplementary figures and images for: BCOR::CREBBP fusion in malignant neuroepithelial tumor of CNS expands the spectrum of methylation class CNS tumor with BCOR/BCOR(L1)-fusion
Source: Acta Neuropathol Commun. 2024 Apr 18;12:60. doi: 10.1186/s40478-024-01780-5 (PMC11025138; doi:10.1186/s40478-024-01780-5)

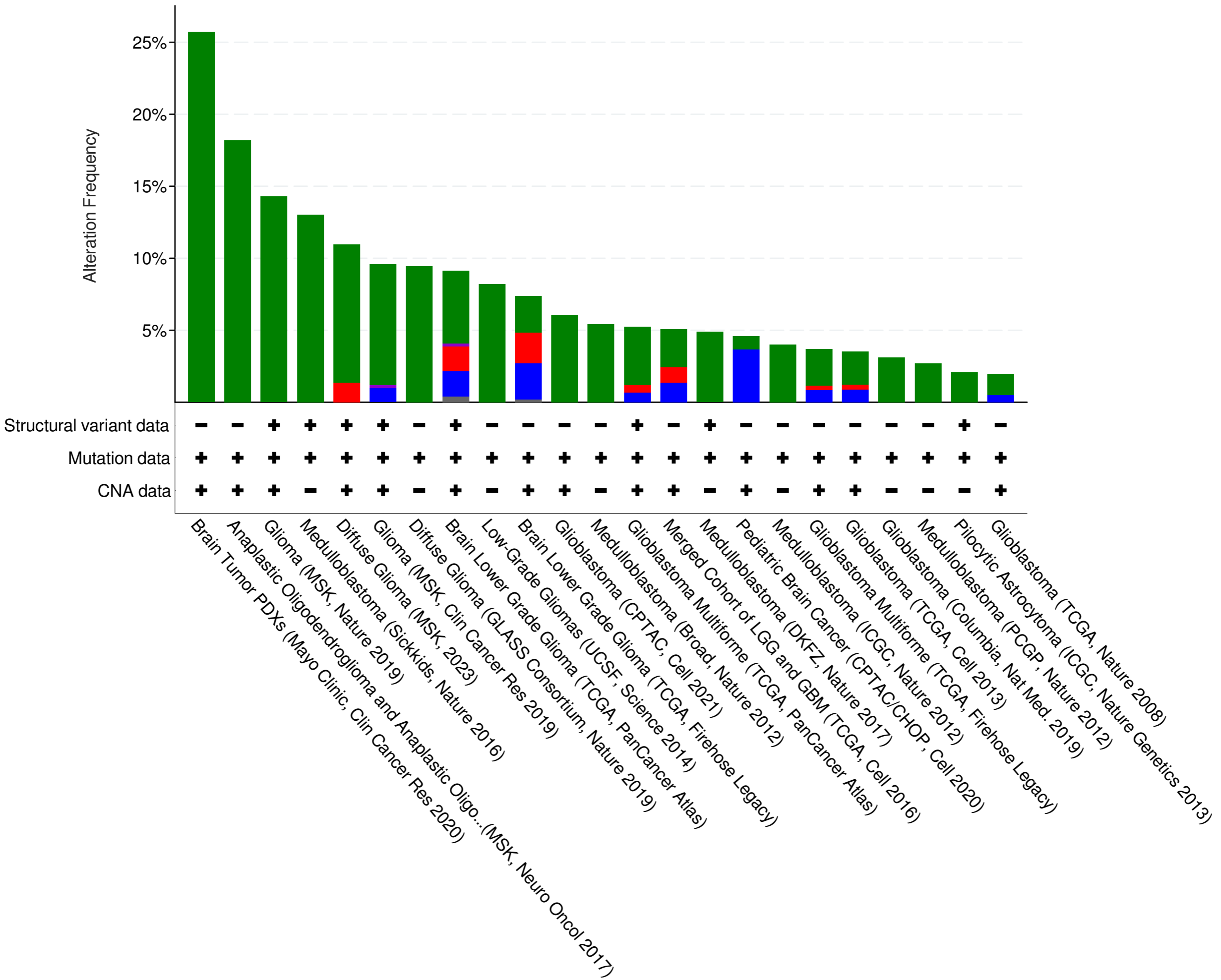

Supplement: Supplementary file 1 — Supplementary Material 1 [file 40478_2024_1780_MOESM1_ESM.pdf]
